# American Academy of Dental Science

**Published:** 1896-05

**Authors:** 

**Affiliations:** American Academy Dental Science


### AMERICAN ACADEMY OF DENTAL SCIENCE.

THE regular monthly meeting of the American Academy of Dental Science was held at Young's Hotel, Boston, Wednesday, December 24, 1895, at 6 o'clock. A talk upon Materia Medica was given by Dr. E. C. Briggs, Boston.

(For Dr. Briggs's paper, see page 294.)

### DISCUSSION.

*Dr. Bradley.*—I have noted down one or two things that Dr. Briggs mentioned, and one is the use of bicarbonate of soda. I have used bicarbonate of soda for some time in cases of sensitiveness around the necks of teeth, but in some cases a patient would complain of a very severe pain when the soda was first applied, and so severe was this pain that the remedy was thought to be worse than the disease.

Within a short time a young lady came to me who had very sensitive cavities on the labial surface of the lower teeth, and as it was such a trial for her to have anything done I recommended that for several days she apply bicarbonate of soda to see if this sensitiveness could not be lessened. She tried it, and returned with the remark that she would prefer to have me excavate the cavities as they were rather than apply bicarbonate of soda about the necks of her teeth. And I would like to ask the essayist if he has met with such cases and how he has dealt with them.

Where there is evidently periostitis, coming on after the filling of a pulpless tooth, I have prescribed a slight cathartic in the first stage, and if there is no beneficial effect from that, I have then prescribed a pill made of one grain of opium and two grains of camphor. I invariably divide the pill and give the patient one-half the quantity to be taken when in bed and prepared to go to sleep; if they are not asleep in one hour they must take the other half of the pill, and I must say that I have had very satisfactory results from the use of this opium and camphor pill. One case in which it worked admirably was that of a young boy, twelve years of age, in whose case it was necessary to devitalize a sixth-year molar. After treating the tooth I filled it, and apparently everything was satisfactory, and he was dismissed. The next day was a wet one and it seemed to strike him as a good day to go fishing. He came home thoroughly wet, with a sore throat as the first symptom, and during the night his tooth began to ache, and by morning a very severe periostitis had developed. I tried to subdue it by external applications, the capsicum plaster, etc., but nothing seemed to relieve him until I gave him this opium and camphor pill. I have not seen him since, but some of the members of the family say that he went to sleep that night and had no further trouble from the tooth.

I shall try the effects of antikamnia in my practice in cases of periostitis which I am unable to subdue by local applications.

*Dr. Piper.*—There is one product of coal-tar that Dr. Briggs did not refer to which I rather expected and hoped he would mention, and that is ammanol.

I have found it to work where the other sedatives did not seem to do much good. Not long ago I had a case of severe pericementitis, and I gave the patient phenacetin in five-grain doses, which she continued taking every half-hour until twenty grains had been taken, and was not relieved. When I saw her, the next day, she was in bed. I prescribed ammanol in ten-grain doses, and she afterwards told me that she seemed to be relieved almost immediately on taking it, and that the pain left in less than twenty minutes. It is probably phenacetin or acetanilide, combined with ammonia or some stimulant, and seems to be more safe to use than phenacetin alone, and the after-effect is more pleasant.

I have been so much pleased with the action of ammanol that I wish some one else would use it.

*Dr. Cooke.*—Perhaps Dr. Briggs would like to say something before we pass this subject.

*Dr. Briggs.*—I don't know that I have anything special to add to what I mentioned at first. In regard to the pain produced by the application of the bicarbonate of soda, spoken of by Dr. Bradley, of course it would not be wise to persist in the use of it; if you find it acts in that way, you have simply to do the best you can with the case as it is presented to you.

This ammanol is a combination of one of the products of coal-tar, all of which have a tendency to depress the circulation, with a stimulant which reduces the liability to danger and makes the drug more effective, and this is the result produced by combining caffeine with acetanilide in antikamnia. Ammonia with phenacetin in ammanol makes another happy combination.

The one point which I would like to emphasize is the improvement which you will be almost certain to attain in your treatment by splitting up your dose. If you know the dose to be a full one, I advise you to split it up, and I think almost all drugs, if a certain dose is what you want to give in twenty-four hours, will work very much better in every way, if you divide it up and spread it over the twenty-four hours in small doses, you do not lose the therapeutic effect and you do lose the toxic effect.

The original nitrous oxide effect was produced by stopping the supply of oxygen,—it was choking a man, so to speak, without preventing the elimination of the $CO_2$, and for that reason has been claimed to be nothing but asphyxia.

I do not regard that as now tenable. I think that there is a true anæsthesia, and experiment will show that to be the effect. The compound oxygen preparations that are used for general tonic stimulants in cases of dynamic fevers, heart-failures, etc., are, most of them, combinations of nitrous oxide with oxygen, and they can, all of them, I think, be taken with considerable anæsthetic effect. I tried a compound oxygen preparation a very short time ago, and two long breaths made me nearer complete anæsthesia than I have ever been with chloroform in quite a good-sized dose, and more so than I have ever been able to get with bromide of ethyl on myself. I am not a very strong advocate of the bromide of ethyl; I have not found it very reliable. Some patients would go into the anæsthesia very quickly and nicely, while with others I could not seem to manage to obtain a satisfactory degree. That was one of those drugs which fell into discredit because of extraneous circumstances connected with cases in which it was administered. Two prominent surgeons used it and had a death. Dr. Levis, in Philadelphia, gave it to a patient who had pulmonary trouble, and the patient died; he called it the effect of bromide of ethyl; J. Marion Sims had one of those long, trying operations on the uterus, and the patient died several hours after the operation, and that was attributed to the bromide of ethyl, consequently it was discredited, and has never been very highly regarded by the profession on account of these accidents, which were really no fault of the drug itself. This ought to bring prominently before us the importance of great care in giving a drug for the production of anæsthesia. If you give it in a definite dose as you would any other medicine, there is no reason why one should not get the result which repeated experiments have shown to be the effect of such a dose. You would not expect to throw a gallon of a thing at a man, and that he would absorb just what was good for him and no more. It has been just this carelessness in the matter of the dose of these petroleum compounds that has discredited them: antipyrin was given in twenty-grain doses until injurious effects were reported from several different quarters; but when they reduced the dose it was found to work satisfactorily. Chloral was first used recklessly, and several fatalities resulted before the dose was brought down to what it should be, and so I think that it is better to try a small dose first and perhaps repeat it rather than give a maximum dose and expect that a man will absorb only what is good for him.

I do not know how the society stands on the subject of anæsthesia. We had a controversy a while ago in which the claims of the two aspirants for the honor of its discovery were presented by their friends, but I do not know who established the strongest claim. I thought I would simply state to the society that I know where there is an oil portrait of Dr. Horace Wells, probably the only one in existence. It was painted at the time he was living in Hartford, and I do not know the circumstances, but rather think that the sitting was secured and the picture bought by some friend of the family, and it would seem as though it ought to be in the possession of the society on account of the strong claims that he had to being the discoverer of anæsthesia. I speak of it simply to put it on record that there is such a portrait in existence, and that it can be bought.

Under the head of "Presentation of Specimens," a model of an automatic mallet, which was something of a novelty, was presented by Dr. Belyea. It was really two mallets in one, both ends being utilized, and, in addition to that, each of the mallets had a direct action and a back action. Of course, the direct action and the back action cannot be used at the same time, but there are times when the back action is much wanted. The chief advantage of the instrument is the time saved in changing points.

WILLIAM H. POTTER, D. M. D.,
*Editor American Academy Dental Science.*

